# Recurrent Bartholin Abscess Resulting in Rare Suspected Recto-Bartholin Fistula: A Case Report

**DOI:** 10.7759/cureus.105003

**Published:** 2026-03-10

**Authors:** Chloe Guidry, Nabil Boutros

**Affiliations:** 1 Emergency Medicine, University of Tennessee Health Science Center (UTHSC) Nashville - Saint Thomas Health, Murfreesboro, USA; 2 Emergency Medicine, Ascension Health, Murfreesboro, USA

**Keywords:** bartholin abscess, bartholin’s gland, fistula, rectal fistula, vulvar pain

## Abstract

This case involves a female patient with a history of recurrent Bartholin abscesses who presented to the emergency department with complaints of purulent drainage from her anus. We present a case of a suspected recto-Bartholin fistula that was identified and initially treated in the emergency department, with the patient ultimately being discharged to outpatient follow-up. A recto-Bartholin’s fistula is an exceptionally rare complication of a Bartholin abscess, and very few have been reported in the medical literature. The article serves primarily to provide education surrounding the presentation, diagnosis, treatment, and rare potential complications of Bartholin abscesses.

## Introduction

The Bartholin glands, or greater vestibular glands, are small structures located bilaterally at the posterior vaginal introitus that primarily function to produce a mucoid secretion that aids in vaginal and vulvar lubrication. The glands connect to the posterolateral aspect of the vaginal orifice via a small duct [1]. Bartholin gland cysts are common in women of reproductive age and account for approximately 2% of all gynecologic visits every year [2]. They develop from duct obstruction leading to mucous accumulation and gland distension. This alone typically results in a painless vaginal mass. However, secondary infection of a Bartholin gland or Bartholin cyst can cause severe pain and swelling that may impair activities such as walking, toileting, and sexual intercourse. Bartholin abscesses are almost three times more common than cysts alone, developing in approximately 2% of women at some point in life [3]. 

The patient in this case suffered from recurrent Bartholin cyst abscesses that ultimately resulted in a suspected recto-Bartholin fistula: an abnormal epithelialized communication between a Bartholin abscess and the rectum. To our knowledge, fewer than five recto-Bartholin fistulas have been documented in the medical literature [4]. As such, there is a paucity of knowledge on how best to manage these complicated cases in the emergency department setting. 

## Case presentation

This patient is a 34-year-old female with a past medical history significant for multiple sclerosis (MS) and at least two prior Bartholin abscesses who presented to the emergency department at our community hospital with concerns that she may have again developed a Bartholin cyst abscess. She stated that three days prior to this presentation, she had developed pain in her rectum. She also reported chills as well as tenderness, swelling, and drainage from the right side of her vulva. Of note, the patient expressed that she also had purulent drainage from her anus when she applied pressure to the right side of her vulva. 

Upon admission to the emergency department, the patient was found to be well-appearing and nontoxic. Her vitals were within normal limits. On physical exam, there was an area of erythema and fluctuance noted in the posterolateral aspect of the right labia majora, which did not extend to the perineum. This area was significantly tender to palpation. She had no active bleeding or discharge from the vulva or from the rectum on digital rectal exam. No fistulous os was identified on visual inspection. 

Laboratory studies were performed and were significant for a white blood cell count of 12.2 x 10^3/mm³. Other laboratory measurements were reassuring, without renal dysfunction, clinically significant metabolic derangement, electrolyte abnormality, or anemia. The serum pregnancy test was negative. Table 1 provides a complete list of laboratory values obtained and reference ranges.

**Table 1:** Laboratory findings Table of laboratory findings for basic metabolic panel (BMP), complete blood count (CBC), and miscellaneous tests with corresponding reference ranges. BUN: blood urea nitrogen; eGFR: estimated glomerular filtration rate; AGAP: anion gap; WBC: white blood cells; RBC: red blood cells; Hgb: hemoglobin; HCT: hematocrit; MCV: mean corpuscular volume; MCH: mean corpuscular hemoglobin; MCHC: mean corpuscular hemoglobin concentration; RDW: red cell distribution width

Test	Result (* If abnormal)	Normal Range
Basic Metabolic Panel		
Sodium	138 mmol/L	136 - 145 mmol/L
Potassium	3.3* mmol/L	3.5 - 5.1 mmol/L
Chloride	109* mmol/L	98 - 107 mmol/L
CO_2_	19* mmol/L	22- 29 mmol/L
BUN	8 mg/dL	7 - 26 mg/dL
Creatinine	1.0 mg/dL	0.6 - 1.1 mg/dL
eGFR	76 mL/min/1.73 m²	>=60 mL/min/1.73 m²
Glucose	94 mg/dL	70 - 105 mg/dL
Calcium, serum	9.0 mg/dL	8.4 - 10.2 mg/dL
AGAP	10 mmol/L	6 - 17 mmol/L
Complete Blood Count		
WBC	12.2 x10^3/mm^3^*	4.8 - 10.8 x10^3/mm^3^
RBC	4.25 x10^6/mm^3^	4.20 - 5.40 x10^6/mm^3^
Hgb	13.2 gm/dL	12.0 - 16.0 gm/dL
Hct	38.6%	35.0 - 47.0%
MCV	90.8 fl	78.0 - 98.0 fl
MCH	31.1 pg	26.0 - 34.0 pg
MCHC	34.2 gm/dL	32.0 - 36.0 gm/dL
RDW	12.1%	11.5 - 14.5%
Platelet	194 x10^3/mm^3^	130 - 400 x 10^3/mm^3^
Ancillary Tests		
Pregnancy, serum	Negative	Negative

**Figure 1:**
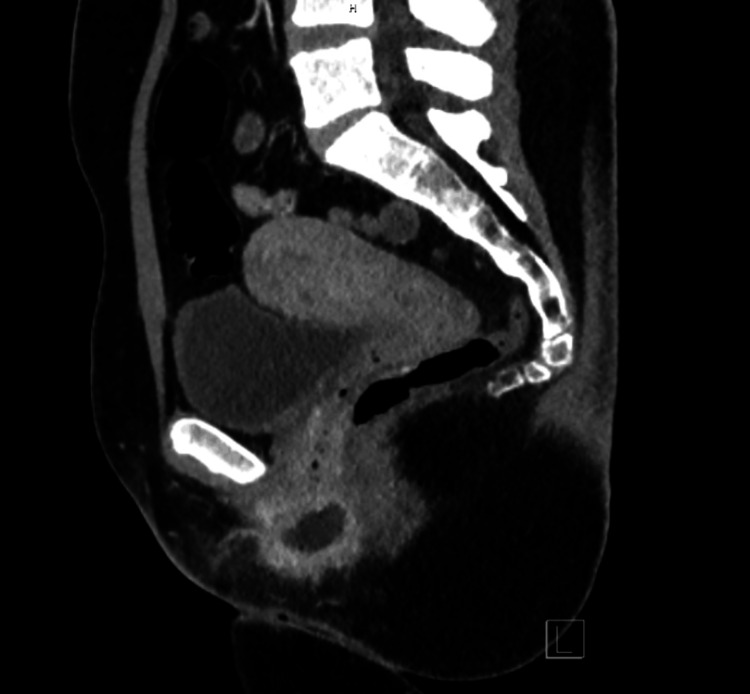
Sagittal image of abdominopelvic CT Showing low-density fluid tracking posteriorly and superiorly from a Bartholin's abscess, along the right side of the distal rectum and anal verge.

The patient also underwent a CT scan of the pelvis with intravenous contrast. Imaging findings included a Bartholin gland abscess along the right posterolateral vulva containing fluid, debris, and internal foci of gas. The abscess measured approximately 30 x 20 x 19 mm. Additional findings include peripherally enhancing fluid extending along the right labia majora and minora, reflecting cellulitis of the right vulva. Finally, CT imaging showed circumferential wall thickening of the lower rectum and anus with fluid tracking posteriorly and superiorly from the Bartholin abscess along the right side of the distal rectum and anal verge (Figure 1). Though no clear communication with the anorectal lumen was seen, the reporting radiologist noted that a fistula cannot be excluded, as the lower rectum and anus were collapsed. Given the patient's reported rectal pain and purulent discharge from the patient's anus, these CT findings raise a strong suspicion for a recto-Bartholin fistula. 

The patient was treated symptomatically in the emergency department with intravenous antiemetics and analgesics. Incision and drainage with placement of a Word catheter was performed. Antibiotic therapy with oral Augmentin and Flagyl was initiated. Of note, on previous visits where the patient had been diagnosed with a Bartholin abscess, the patient was treated in a similar manner with incision and drainage with Word catheter placement and with oral Flagyl. The patient’s presentation, imaging findings, and laboratory results were discussed with the on-call gynecologist. As the patient was afebrile, hemodynamically stable, and well-appearing without significant laboratory abnormalities or evidence of necrotizing peritoneal infection, both the gynecologist and emergency medicine provider felt that the patient could be safely discharged on oral antibiotics. She was instructed to seek further evaluation and treatment from her gynecologist as well as a general surgeon in the outpatient setting. Unfortunately, we have not been able to successfully contact the patient to assess the efficacy of treatment and if further interventions are required. To our knowledge, the patient has not undergone any definitive surgical management at this time. 

## Discussion

A review of the literature reveals that as of 2021, there were fewer than 10 reported cases of rectovaginal fistulas resulting from a Bartholin cyst abscess [5]. There are even fewer reported cases of recto-Bartholin fistulas. Repeated occurrences of abscesses, delayed drainage, and immunocompromise may increase the risk for fistula formation, which is thought to be the result of chronic inflammation. According to a 2013 study, 81% of recurrent abscesses will recur ipsilaterally, and most within 32 months [6]. A Bartholin cyst abscess is most frequently diagnosed via physical examination, which may reveal a unilateral medially protruding mass in the posteromedial vulva with overlying erythema and surrounding induration or fluctuance [3]. The mass is often tender to palpation and may express purulent discharge. Diagnosis does not typically require laboratory or imaging studies unless a complicated process, such as deep-space infection, is clinically suspected. If a fistula is suspected, as in this case, further evaluation with a CT of the pelvis with intravenous contrast is recommended. If the diagnosis is still unclear, the presence of a fistula may be further evaluated with an MRI or an examination under anesthesia to make a definitive diagnosis. 

Treatment 

The treatment of a Bartholin’s cyst or abscess depends on the patient’s presenting symptoms. Asymptomatic cysts typically do not require treatment. Abscesses that are freely draining may be managed conservatively with analgesics and sitz baths. Simple incision and drainage is not recommended due to high rates of recurrence [7]. If drainage of an abscess is indicated, a Word catheter, a small silicone tube with an inflatable balloon on one end, should be placed into the incised abscess cavity to allow continued drainage. Word catheters should be left in place for at least four weeks to ensure proper tract epithelization and resolution of abscess [8]. A similar procedure using a Jacobi ring may be more accessible in resource-poor emergency departments or rural settings. This technique involves threading a butterfly needle tubing with suture material, then passing the tubing and suture through the abscess cavity. The suture material is tied together, forming a ring. This method reduces the risk for early malposition and is associated with greater patient satisfaction [9]. 

For patients who experience recurrent Bartholin cysts or abscesses, such as the patient described in this case report, surgical management is often recommended. This is most often accomplished via a procedure termed marsupialization and is performed by a gynecologic surgeon. The procedure involves creating an incision lateral to the hymenal ring and suturing the everted edges of the incision onto the epithelial surface with absorbable sutures. It is important to note that a 2014 meta-analysis found no significant difference in rates of recurrence between marsupialization and Word catheter placement [10]. If a fistula is identified, fistulotomy may also be necessary.

Antibiotic therapy should be considered in patients who have failed initial treatment with catheter drainage therapy, those with systemic symptoms such as fever or signs of sepsis, and those with an increased risk for recurrence. However, the optimal choice of initial antibiotic is still controversial. Bartholin gland abscesses are polymicrobial, though *Escherichia coli* is the single most common pathogen identified on culture [6]. *Escherichia coli* was also significantly more likely to be associated with recurrent infections than primary infections [6]. Bartholin’s abscesses are not considered to be exclusively the result of sexually transmitted infections. Given the high rates of polymicrobial infections, antibiotic coverage should initially be broad and be narrowed based on culture results. Flucloxacillin and Augmentin have historically been recommended as first-line options [11]. 

## Conclusions

Recto-Bartholin fistula is a rare complication of Bartholin abscess, but it should remain on the differential when purulent rectal drainage accompanies vulvar swelling. If suspected, CT imaging with intravenous contrast is recommended for further evaluation. If a fistula is identified, a multidisciplinary approach involving gynecological and general surgical services is most appropriate. If CT imaging is inconclusive, as in the case presented here, further evaluation with MRI or examination under anesthesia can provide a definitive diagnosis. Management in the emergency department should focus on initiation of antibiotics, symptom control, and expedited outpatient referral to reduce the likelihood of recurrence and further complications.

## References

[1] Quaresma C, Sparzak PB (2026). Anatomy, abdomen and pelvis: Bartholin gland. StatPearls [Internet].

[2] Marzano DA, Haefner HK (2004). The bartholin gland cyst: past, present, and future. J Low Genit Tract Dis.

[3] Omole F, Simmons BJ, Hacker Y (2003). Management of Bartholin’s duct cyst and gland abscess. American Family Physician.

[4] Kim YS, Han HS, Seo MW, Kim WS, Lee JH, Park NK, Sang JH (2015). Recto-Bartholin's duct fistula: a case report. Gynecol Obstet Invest.

[5] Bensardi FZ, Kabura S, Layla E, El Bakouri A, Bouali M, El Hattabi K, Fadil A (2021). Bartholin's gland abscess a rare cause of rectovaginal fistula: a case report and literature review. Int J Surg Case Rep.

[6] Kessous R, Aricha-Tamir B, Sheizaf B, Shteiner N, Moran-Gilad J, Weintraub AY (2013). Clinical and microbiological characteristics of Bartholin gland abscesses. Obstet Gynecol.

[7] Long N, Morris L, Foster K (2021). Bartholin gland abscess diagnosis and office management. Prim Care.

[8] Carlson K, Wittler M (2026). Bartholin gland cyst. StatPearls [Internet].

[9] Gennis P, Li SF, Provataris J, Shahabuddin S, Schachtel A, Lee E, Bobby P (2005). Jacobi ring catheter treatment of Bartholin's abscesses. Am J Emerg Med.

[10] Bakouei F, Zolfaghari F, Mirabi P, Farhadi Z, Delavar MA (2024). Comparison of word catheter and marsupialization in the management of Bartholin’s glands: a systematic review and meta-analysis. J Obstet Gynaecol Can.

[11] Bhide A, Nama V, Patel S, Kalu E (2010). Microbiology of cysts/abscesses of Bartholin's gland: review of empirical antibiotic therapy against microbial culture. J Obstet Gynaecol.

